# AICAR Administration Attenuates Hemorrhagic Hyperglycemia and Lowers Oxygen Debt in Anesthetized Male Rabbits

**DOI:** 10.3389/fphys.2017.00692

**Published:** 2017-09-13

**Authors:** Yi Huang, Paul H. Ratz, Amy S. Miner, Victoria A. Locke, Grace Chen, Yang Chen, Robert W. Barbee

**Affiliations:** ^1^Department of Emergency Medicine, Virginia Commonwealth University Richmond, VA, United States; ^2^Medical Center of Stomatology, The First Affiliated Hospital of Jinan University Guangzhou, China; ^3^Department of Biochemistry and Molecular Biology, Virginia Commonwealth University Richmond, VA, United States; ^4^Department of Pediatrics, Virginia Commonwealth University Richmond, VA, United States; ^5^Department of Pharmacology and Toxicology, Virginia Commonwealth University Richmond, VA, United States; ^6^Department of Physiology and Biophysics, Virginia Commonwealth University Richmond, VA, United States

**Keywords:** AMPK, glucose transport, glucose oxidation, hemodynamics, ischemia, lactate, oxygen consumption, oxygen delivery

## Abstract

**Background:** Many strategies have been utilized to treat traumatic shock via improved oxygen delivery (DO_2_), while fewer have been used to in an attempt to reduce oxygen demand (VO_2_). The cellular energy sensor 5′ adenosine monophosphate-activated protein kinase (AMPK) has the potential to modulate both whole-body DO_2_ and VO_2_. Therefore, we determined the effect of the AMPK activator AICAR (5-aminoimidazole-4-carboxamide 1-β-D-ribonucleoside) given acutely or chronically on key metabolites, hemodynamics, and oxygen consumption/delivery before and during hemorrhage in anesthetized male rabbits.

**Methods:** Chronically treated animals received AICAR (40 mg/kg/day, IV) for 10 days prior to hemorrhage, while rabbits in the acute study were infused with AICAR (7.5 mg/kg bolus, 2 mg/kg/min infusion) or vehicle (0.3 ml/kg saline bolus, 0.03 ml/kg/min infusion) IV for 2 h prior to severe hemorrhage. Both acutely and chronically treated animals were sedated (ketamine/xylazine cocktail) the morning of the terminal experiment and surgically prepared for hemorrhage, including the implantation of arterial and venous catheters (for blood removal/sampling and drug/vehicle administration) and thoracotomy for implantation of transit-time flow transducers (for cardiac output determination).

**Results:** AICAR given acutely lowered arterial blood glucose and increased blood lactate levels before hemorrhage, and abolished the well-documented hemorrhage-induced hyperglycemia seen in vehicle treated animals. Animals given AICAR chronically had blunted hemorrhage-induced hyperglycemia without prior baseline changes. Chronically treated AICAR animals showed significantly lower lactate levels during hemorrhage. Rabbits receiving AICAR both acutely and chronically experienced similar falls in mean arterial pressure, cardiac output and hence DO_2_ to their vehicle counterparts throughout the hemorrhage period. However, rabbits treated either acutely or chronically with AICAR accumulated lower oxygen deficits and debt during hemorrhage compared to vehicle-infused controls.

**Conclusions:** The oxygen debt data suggest that AMPK activation could decrease trauma associated morbidity and mortality, perhaps by mechanisms related to increased glucose utilization. Additional studies are needed to investigate the effects of AICAR and associated mechanisms of action when given during resuscitation from hemorrhage.

## Introduction

Hemorrhage is responsible for ~40% of all trauma-related deaths (Sauaia et al., 1995), and most occur in the first 6 h following surgery (Shackford et al., 1993). Hyperglycemia is a normal part of the metabolic stress response (Neligan and Baranov, 2013), which was originally thought to be adaptive in hemorrhage (Menguy and Masters, 1978). However, more recent evidence suggests prolonged hyperglycemia is associated with negative outcomes including multiple organ failure (Sperry et al., 2007), and the use of insulin to control hemorrhage-induced increases in blood glucose is not without risk (Finney et al., 2003). The nucleoside analog 5-aminoimidazole-4-carboxamide 1-β-D-ribonucleoside (AICAR) can increase glucose transport into skeletal muscle and therefore lower blood glucose via insulin-independent mechanisms (Krook et al., 2004).

Accumulated oxygen debt has been described as one of the best predictors of morbidity and mortality in trauma (Barbee et al., 2010). The calculation of oxygen deficit (which integrated over time becomes oxygen debt) assumes that the hemorrhage induced reduction in oxygen consumption is due to inadequate oxygen delivery to the mitochondria, leading to a reduction of ATP production. However, oxygen consumption could also decrease via oxygen sparing strategies, reducing whole body oxygen demand. Several such mechanisms have been shown to operate in anoxia tolerant organisms (Hochachka et al., 1996). Notably, activation of AMPK (5′ adenosine monophosphate-activated protein kinase), a key sensor of metabolic stress (Hardie, 2014), regulates cellular metabolism to reduce oxygen consumption, limiting conversion of ATP to AMP. AICAR, an activator of AMPK (Steinberg and Kemp, 2009), has been shown to reduce myocardial infarct size (Paiva et al., 2010), reduce renal fibrosis along with markers of cell stress and apoptosis (Decleves et al., 2014), and reduce both gut damage and lung neutrophil infiltration (Idrovo et al., 2014) in rodent ischemia-reperfusion models. Furthermore, AICAR extends hemorrhagic shock survival times in rats (Sonneborn and Rutten, 2011). Therefore, we examined the metabolic and hemodynamic consequences of both acute and chronic AICAR dosing in a rabbit model of hemorrhagic shock. The rabbit model was used as a source of vessels for later *in vitro* analyses of vascular smooth muscle function in shock (Ratz et al., 2016). Also, the rabbit is a larger animal closer in size to human infants, so compared to rodents, regulation of the cardiovascular system (especially control of heart rate vs. stroke volume in determining cardiac output) is closer to the human system. These experiments were designed as part of a larger planned long-term treatment series (acute and chronic pretreatment, followed by immediate and delayed resuscitation treatment).

## Methods

### Animals

All studies were approved in advance by the Institutional Animal Care and Use Committee (IACUC) of Virginia Commonwealth University and conform to the Public Health Service Policy on Humane Care and Use of Laboratory Animals (revised, 2015) and the National Research Council “Guide for the Care and Use of Laboratory Animals” (Eighth Edition, 2011). Male New Zealand rabbits (range of 2.8–3.6 kg, mean = 3.18 ± 0.23 kg, 12–15 weeks of age, specific-pathogen free) were obtained from Robinson Services, Inc. and maintained in the vivarium at 19–22°C and 12L:12D for at least 6 days prior to experimentation. Animals were individually housed but provided toys as environmental enrichment and fed a combination of pelleted high-fiber rabbit food (Harlan Teklad 2031, approximately one cup/day) and hay (Timothy® approximately one handful of loose hay plus ½ high-fiber hay cube).

### Initial survival surgery

All animals in the chronic AICAR dosing study received an initial minor survival surgery for placement of indwelling arterial and venous catheters for blood sampling and drug infusion. Following an overnight (~12 h) fast, animals were initially sedated with a ketamine/xylazine cocktail (50:5 mg/kg, IM), followed by preparation of surgical sites [clipping followed by depilatory agent (Nair) application and then removal with saline followed by cleaning with an iodine solution and 70% alcohol]. Animals were transferred to a heating pad (water circulated, set at 105° F), and administered a single dose of extended release buprenorphine (Buprenorphine SR-LAB; 0.1 mg/kg sq; Zoopharm; Windsor, CO; http://wildpharm.com/medications/labanimals/item/3-buprenorphine-sr-1ml.html) for intra-and postoperative analgesia. If necessary, anesthesia was maintained with isoflurane (0.5–2.0%, balance oxygen). Following isolation of the right jugular vein, a heparin coated catheter (CBAS-C35; Solomon Scientific; San Antonio, TX) was inserted and advanced to the level of the right atrium for subsequent infusions. The proximal end was then tunneled subcutaneously and externalized at the nape of the neck where the catheter end was secured in a “cap” fashioned from a plastic disc with attached lid. A 24 gauge Jelco catheter (Smiths Medical; Dublin, OH) was inserted into the central ear artery and secured with tape and gauze for later blood sampling. Both catheters were filled with a heparin/dextrose (500 U/ml/50%) solution (Braintree Scientific; Braintree, MA) to preserve patency. Following surgery, all animals received Ringer's solution (10 ml/kg; ½ ip, ½ sq) to prevent postsurgical dehydration. Animals recovered on a heating pad until they had achieved sternal recumbency, and were then returned to the animal vivaria.

### AICAR or vehicle treatment

Three days following the initial surgery, animals were randomly selected to receive daily treatment with AICAR [5-aminoimidazole-4-carboxamide 1-β-D-ribonucleoside, 40 mg/kg/day (Park et al., 2009); Cayman Chemical Company; Ann Arbor, MI] or vehicle (0.9% saline; 1 ml/kg/day) for a period of 10 days. Drug or vehicle solutions were given as a bolus over 0.5–1 min. Immediately before infusion on days 1, 4, 7, and 10, blood was sampled from the Jelco catheter for glucose and lactate levels (ABL90 Flex, 65 μl; Radiometer America Inc.; Westlake, OH).

### Final acute (non-survival) surgery

Following an overnight (~12–15 h) fast on the final day of AICAR or vehicle treatment, animals were sedated with a ketamine/xylazine cocktail (50:5 mg/kg, IM) and temporarily supplemented if needed with isoflurane (0.5–5%, balance oxygen). They were then placed on a heating pad, where the surgical sites were clipped; depilatory agent (Nair) was applied to remove remaining hair. The sites were then cleaned with 70% alcohol. Animals were transferred to a temperature-controlled heating pad set at 39°C; surgical towels were added or removed to keep core temperature from deviating more than 0.5°C until the initiation of hemorrhage, at which time no additional attempts were made to control body temperature, other than feedback from the heating pad. A lateral ear vein was cannulated (Jelco 23 gauge, ½″ radiopaque IV catheter), and the animal was transitioned to Alfaxan (alfaxalone, concentration of 10/mg/ml, Jurox; London, UK; http://alfaxan.co.uk/; average dose of ~100 μg/kg/min, range of 3–200 μg/kg/min). Tracheostomy was performed using a size 3.5 F endotracheal tube (Kendall Healthcare Corp; Mansfield, MA) to initially facilitate breathing and for later connection to a ventilator (see below). Polyethylene catheters (PE-90) were placed into the terminal aorta via the left femoral artery (LFA), terminal inferior vena cava via the left femoral vein (LFV), ascending aorta via the right carotid artery (RCA), and the right atrium via right jugular vein (RJV). The position of all catheter tips was confirmed at necropsy. The LFA was used to monitor arterial pressure (Transpac IV disposable pressure transducers; ICU Medical; San Clemente, CA 92673) and collect arterial blood samples, whereas the LFV was used for pre-hemorrhagic IV treatment infusion [(AICAR, bolus of 7.5 mg/kg, followed by an infusion of 2 mg/kg/min (Christopher et al., 2006; Rantzau et al., 2008) or vehicle, 0.9% NaCl (Baxter Healthcare Corp; Deerfield, IL); bolus of 0.3 ml/kg followed by an infusion of 0.03 ml/kg/min] for 2 h before hemorrhage or infusion of crystalloid (Ringer's solution; Baxter Healthcare Corp; Deerfield, IL) as needed to support mean arterial blood pressure (MAP) during hemorrhage (see hemorrhage protocol below). Bolus solutions were given over a period of 0.5 min. The RCA was used for blood removal while the RJV was used for central venous blood sampling. A parasternal thoracotomy, considered a less invasive alternative (and therefore refinement) to a median sternotomy (Luciani and Lucchese, 2013), was performed by cutting the first and second ribs near the insertion to the sternum and rapid cauterization of any cut vessels.

### Hemodynamics and sampling

Each animal was placed on a ventilator [SAR-1000; CWE, Inc; Ardmore, PA; initial rate of ~0.33 Hz, end-inspiratory pressure of 8 cm H_2_O (i.e., pressure-mode ventilation), sigh breath of 20 cm H_2_O (every 20 breaths)], and a transit-time flow transducer (MA4PSB; Transonic Systems, Inc; Ithaca, NY) was placed on the ascending aorta for estimation of cardiac output (CO). The transducer was connected to a flow meter (T403, TS420 module; Transonic Systems Inc., Ithaca, NY). The blood pressure signals were first amplified, while the phasic flow signal was sent directly to an analog-digital board (MP150; Biopac Systems, Inc.; Goleta, CA). All data was collected at 400 Hz. The upstroke of the flow signal was used to calculate heart rate (HR), while the arterial blood pressure signal was filtered to estimate mean arterial pressure (MAP). The data was displayed and saved using Acknowledge™ software (version 4.2; Biopac Systems, Inc.; Goleta, CA). Ventilatory end-inspiratory pressure and/or frequency were used to adjust arterial PCO_2_ to ~40 mm Hg prior to hemorrhage. Blood sampling occurred every 10 min for 2 h before hemorrhage during AICAR or saline infusion. Femoral artery or jugular vein catheter sampling was used to measure blood gases, electrolytes and metabolites (glucose and lactate; ABL90 Flex, 65 μl; Radiometer America Inc.; Westlake, OH). For more complete model characterization, catecholamine (epinephrine, norepinephrine) levels were determined in several animals using a 2-CAT (A-N) research ELISA kit (Labor Diagnostika Nord, Nordhorn, Germany). Samples were taken at –180, 0, 15, and 65 min during surgery and hemorrhagic shock, prepared, and results calculated per company instructions (http://www.ldn.de/sites/default/files/downloads/BA%20E-6500.pdf). Briefly, sodium metabisulfite (final concentration 4 mM) and EDTA (final concentration 1 mM) was added to all samples to prevent catecholamine degradation and blood coagulation, which were subsequently centrifuged at 2,000 g for 15 min at 4°C. The plasma was analyzed using a microplate reader (ELx800; BioTek, Winooski, VT) with the filter set for excitation at 450 and emission at 630 nm. Maximum blood sampling for all of these tests did not exceed 5% of the total blood volume and did not vary more than ~1% of the total blood volume within animals.

### Hemorrhage protocol

Animals were hemorrhaged from the carotid artery by withdrawing blood with a programmable syringe pump (PHD 2000 Series, Harvard Apparatus, Holliston MA) at three sequential rates (3, 2, and 1 ml/kg/min). Rates were lowered from the highest rate of withdrawal when 20 ml of blood (~10% blood volume) was removed or when MAP was less than 35 mm Hg. Blood withdrawal stopped when MAP reached 30 mm Hg, and restarted when MAP exceeded 35 mm Hg for more than 5 s. If MAP fell to less than 25 mm Hg, Ringer's solution without added glucose (Baxter Healthcare Corp; Deerfield, IL) was infused at 3 ml/kg/min and was terminated when MAP exceeded 30 mm Hg. This resulted in mean arterial pressure “clamping” at ~30–35 mm Hg. To prevent further pressure deterioration during hemorrhage without encouraging atelectasis, sigh frequency was switched from every 20th to every 200th breath in all hemorrhaged animals just before the onset of hemorrhage. Blood gas/metabolite sampling occurred as listed above (during AICAR or vehicle dosing), immediately before, 5 min after hemorrhage and every 10 min thereafter until 75 min, which represented the end of the experiment. Oxygen delivery (DO_2_) was calculated as the product of the CO and arterial oxygen content. Oxygen consumption (VO_2_) was calculated using the reverse Fick principle; the position of the jugular vein catheter tip was confirmed as in or near (within 1 cm) the right atrium at necropsy. Oxygen deficit was calculated by subtracting the VO_2_ at a given time point in hemorrhage from the baseline VO_2_. Oxygen debt was calculated as the sum of the oxygen deficit for a given time period plus all previous time periods following baseline (i.e., the oxygen deficit integral). Sham animals received all surgical instrumentation and were followed for the same time period as controls but were not infused or hemorrhaged.

### Data analysis and statistics

All data is presented as the mean ± the standard error of the mean (SEM), analyzed using Graph Pad Prism 6.0 software. Two-Way Analysis of variance (ANOVA) with repeated measures was performed to compare the relevant groups. A *P*-value of <0.05 was considered statistically significant, except when adjusted using the Bonferroni correction (multiple *t*-tests involving catecholamine analysis). Chronic controls (i.e., animals given 1 ml/kg/day saline) exhibited hemorrhage responses similar to acute controls and are not shown here for sake of brevity and to facilitate comparisons of acute and chronic AICAR treatment. Changes described in hemorrhage responses refer back to the zero time point (i.e., the last data point recorded before starting blood withdrawal) with the exception of oxygen deficit and debt calculations. In this case, variability in the oxygen consumption measurements between groups led us to combine the oxygen consumption measurements for both 10 and 0 min before hemorrhage as a baseline for subsequent deficit (and hence debt) calculations. Sham data are reported to show stability of the preparation and for comparisons to both treatment groups (i.e., AICAR and vehicle) when those groups showed similar responses.

## Results

### Effect of acute and chronic AICAR administration on hemodynamics prior to and during hemorrhage

**Figure 1:**
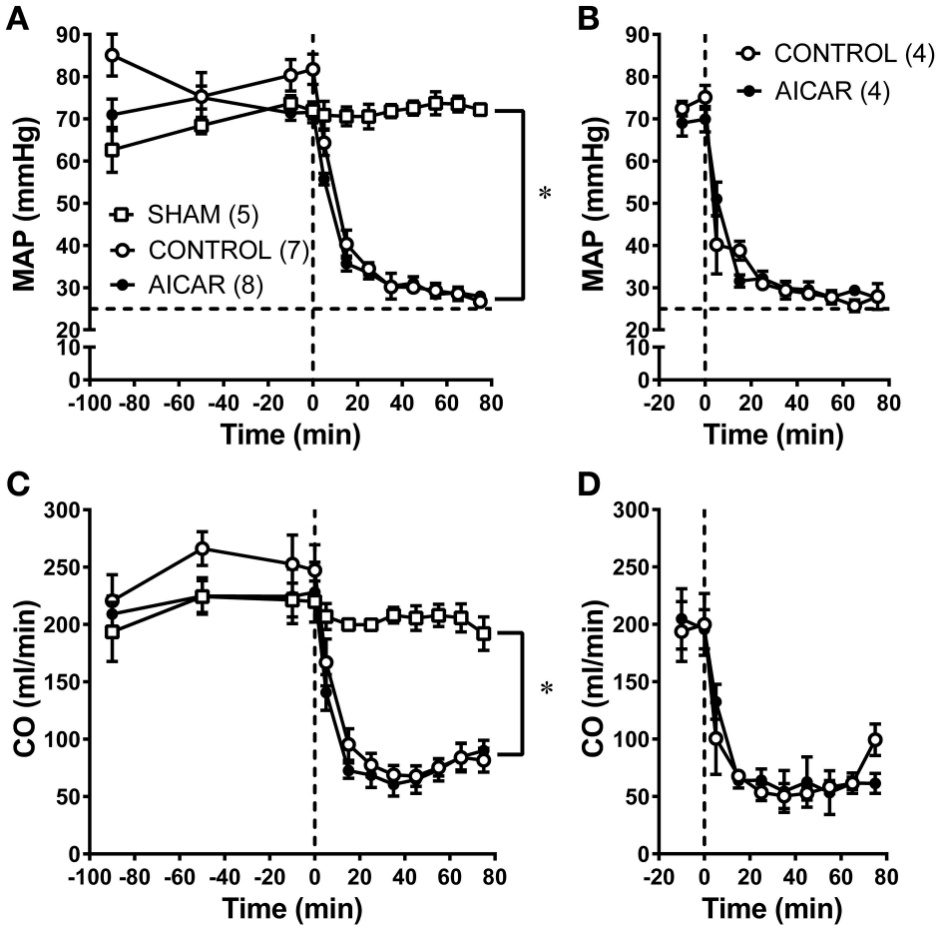
Comparison of hemodynamics [MAP **(A,B)** and CO **(C,D)** in rabbits treated with AICAR acutely (2 h; **A,C**) or chronically (10 days) during baseline and hemorrhage compared to controls (given vehicle) and sham animals (acute studies only—instrumented but not hemorrhaged). Horizontal dashed lines in **(A,B)** represent the lower limit (i.e., 25 mm Hg) for the hemorrhage pressure clamp; vertical dashed lines in **(A–D)** represent the onset of hemorrhage (see Section Methods)]. ^*^P < 0.05 for ANOVA group effects for control and AICAR-treated compared to sham animals, **(A,C)**. Number of animals/group are indicated in parentheses (#'s for **C,D** equal those for **A,B**).

The effects of acute (2 h) or chronic (10 day) AICAR treatment on selected hemodynamic variables is shown in Figure 1. There was no effect of acute (Figures 1A,C) or chronic (Figures 1B,D) AICAR treatment compared to vehicle treated animals on basic hemodynamics (MAP or CO) before the onset of hemorrhage. Furthermore, AICAR treatment was associated with similar and substantial (i.e., significant time effect or change over time, Figures 1A–D) decreases in both MAP and CO following the onset of hemorrhage compared to vehicle treated animals. However, the MAP and CO changes were significantly different compared to sham animals (Figures 1A,C). Sham animals had no significant changes in hemodynamics throughout the experiment (Figures 1A,C). Due to modest, non-significant increases in heart rate during hemorrhage, we decided to monitor plasma catecholamine changes during hemorrhage in several rabbits (*n* = 3/group) receiving acute AICAR, vehicle or sham treatment to assess the degree of sympathetic activation. Sham animals showed only modest increases in plasma epinephrine and norepinephrine during the hemorrhage period, peaking at 0.84 ± 0.46 and 1.2 ± 0.29 ng/ml respectively at 65 min. The vehicle and AICAR treated animals showed similar and immediate increases in plasma epinephrine and so were combined (*n* = 6), peaking at 15 min (mean combined level of 5.6 ± 1.2 ng/ml; significantly elevated compared to sham) and remaining elevated throughout hemorrhage. These same animals exhibited roughly linear increases in norepinephrine, with peak levels averaging 4.5 ± 0.9 ng/ml (AICAR) and 4.5 ± 0.5 ng/ml (vehicle) at 65 min of hemorrhage (significantly elevated compared to sham; data not shown). Thus, our hemorrhage protocol caused sympathetic nervous system activation.

### Effect of acute and chronic AICAR administration on oxygen consumption, delivery, deficit, and debt

**Figure 2:**
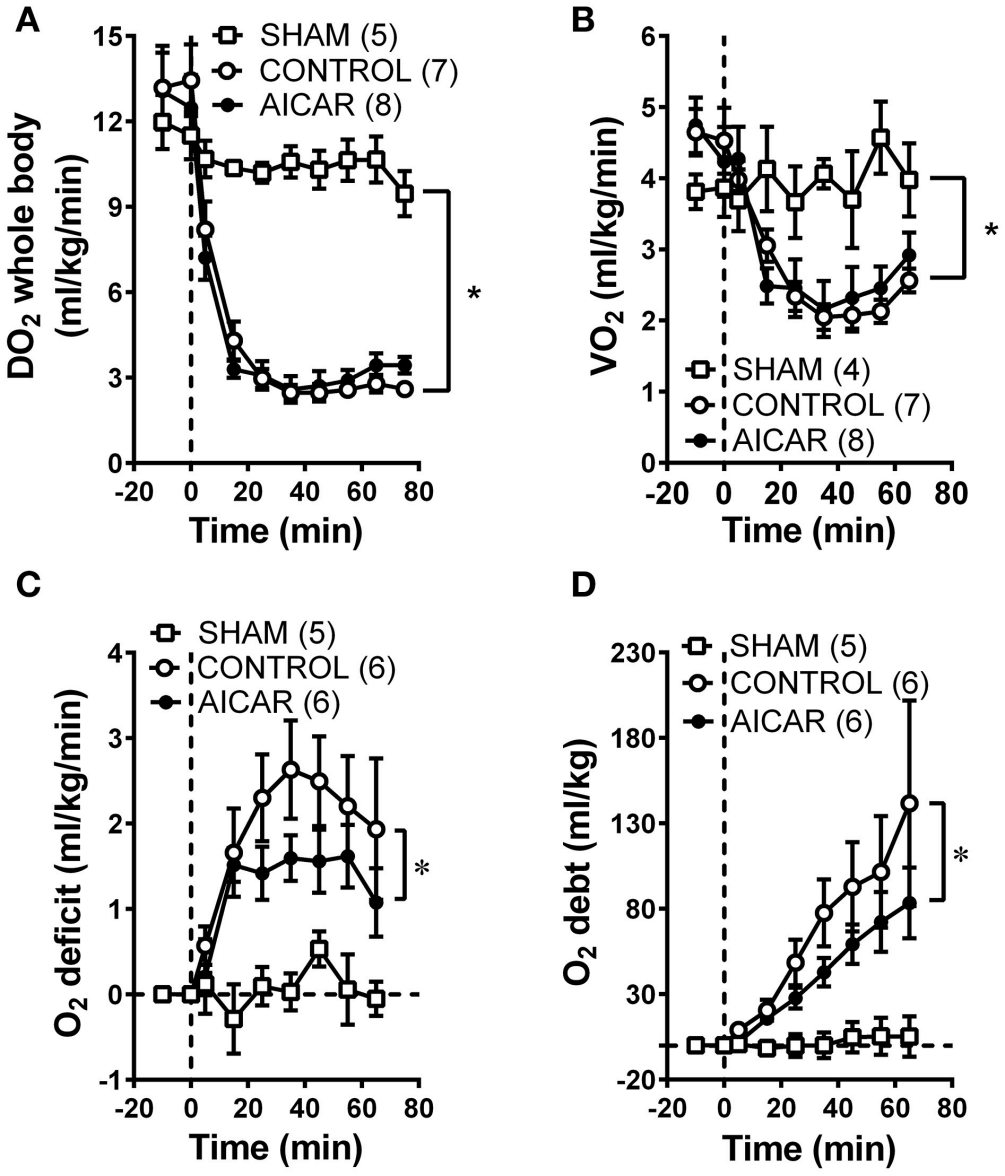
Comparison of whole animal oxygen delivery **(A)**, oxygen consumption **(B)**, oxygen deficit **(C)**, and accumulated oxygen debt **(D)** at baseline and during hemorrhage in animals treated acutely with AICAR compared to controls (given vehicle) and sham animals (instrumented but not hemorrhaged). ^*^P < 0.05 for ANOVA group effects for control and AICAR-treated compared to sham animals, Figures 2A,B; group effect, AICAR compared to controls, **(C,D)**.

**Figure 3:**
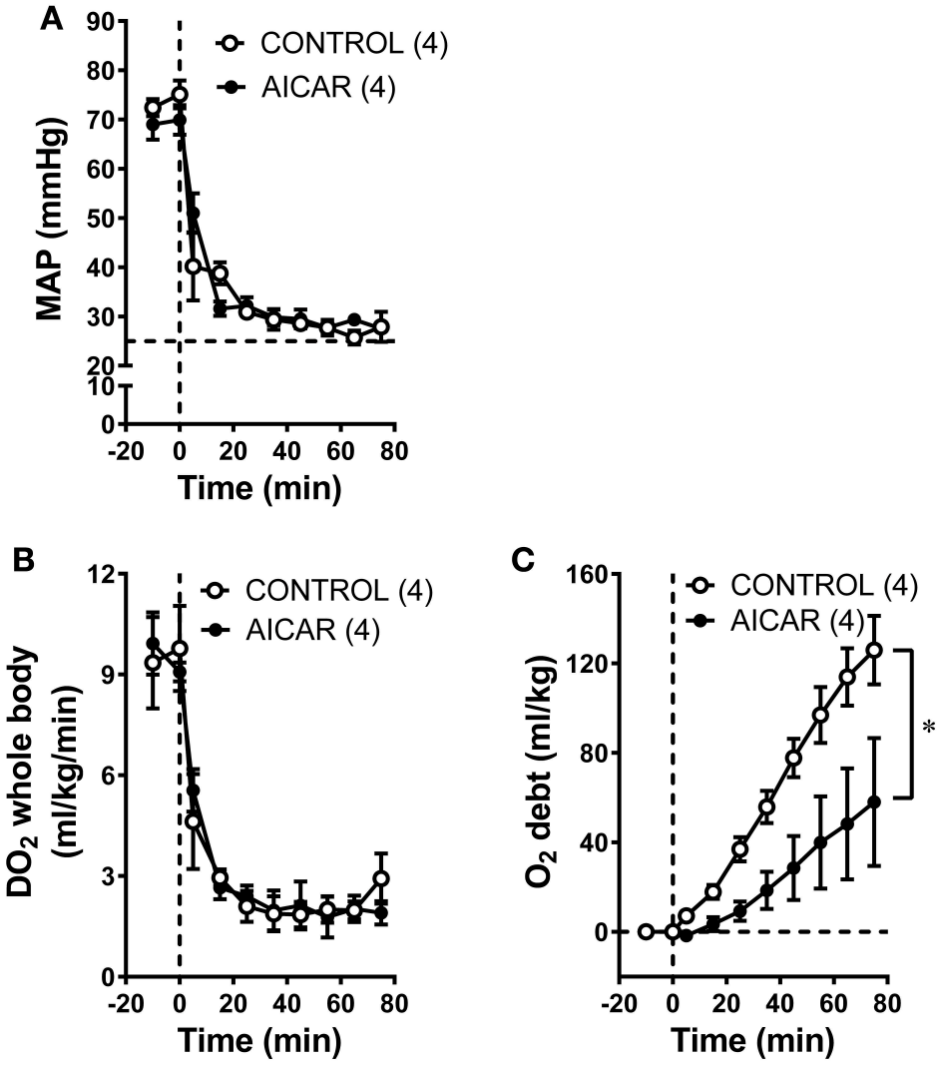
Comparison of MAP **(A)**, whole animal oxygen delivery **(B)**, and accumulated oxygen debt **(C)** at baseline and during hemorrhage in animals treated chronically with AICAR compared to controls (given vehicle). ^*^P < 0.05 for ANOVA group effect, AICAR compared to controls, **(C)**.

As expected from the nearly identical decreases in both MAP and CO in acutely treated rabbits, oxygen delivery (i.e., the product of cardiac output and oxygen content) demonstrated similar declines in both acutely-treated AICAR and control animals following the onset of hemorrhage (Figure 2A, significant time effect), that were nevertheless significantly different from the responses seen in sham animals (Figure 2A, significant group effect). The oxygen consumption response to hemorrhage was more complex (Figure 2B). Baseline values for oxygen consumption were not significantly different among groups. Both control and AICAR (acute-treatment) animals responded to hemorrhage with similar drops in oxygen consumption for the first 35 min following the onset of volume removal (Figure 2B, significant time effect) that were lower than oxygen consumption seen in sham animals (Figure 2B, significant group effect). Both groups then demonstrated slight recoveries in oxygen consumption; rabbits receiving acute treatment with AICAR showed slightly (but not significantly) greater recovery in oxygen consumption toward baseline values. This slightly greater recovery of oxygen consumption led to a significant reduction in oxygen deficit in AICAR treated, compared to control animals (Figure 2C, two-way ANOVA group effect). Likewise, oxygen debt (the accumulated deficit over time) was also significant lower in AICAR treated animals when compared with controls (Figure 2D, two-way ANOVA group effect). Sham animals showed no significant alterations in oxygen delivery or consumption during the experiment (Figures 2A,B), and as a result, negligible accumulations of oxygen deficit and debt (Figures 2C,D). Despite nearly identical hemodynamic changes following the onset of hemorrhage in animals chronically treated with AICAR compared to controls (Figures 3A,B), oxygen debt was reduced even more (by ~½ at the end of hemorrhage) in AICAR treated animals compared to chronically treated vehicle controls (group effect, Figure 3C). This was due to slightly smaller decreases in oxygen consumption in the AICAR treated animals (Table 1), leading to smaller deficits (Table 2) and debt (Figure 3C).

**Table 1:** Oxygen consumption (ml/kg/min) over time in animals chronically treated with vehicle vs. AICAR.

**Time (min)**	**-10**	**0**	**5**	**15**	**25**	**35**	**45**	**55**	**65**	**75**
Control	3.5 ± 0.5	3.7 ± 0.2	2.7 ± 0.4	2.5 ± 0.2	1.8 ± 0.4	1.6 ± 0.4	1.6 ± 0.3	1.7 + 0.3	1.8 + 0.2	2.4 + 0.5
AICAR	3.0 ± 0.3	2.4 ± 0.4	2.9 ± 0.5	2.2 ± 0.3	2.0 ± 0.3	1.6 ± 0.5	1.8 ± 0.6	1.5 ± 0.6	1.8 ± 0.4	1.7 ± 0.3

**Table 2:** Oxygen deficit (ml/kg/min) over time in animals chronically treated with vehicle vs. AICAR.

**Time (min)**	**-10**	**0**	**5**	**15**	**25**	**35**	**45**	**55**	**65**	**75**
Control	0 ± 0	0 ± 0	0.9 ± 0.3	1.1 ± 0.3	1.8 ± 0.3	2.0 ± 0.4	2.0 ± 0.2	1.9 ± 0.3	1.9 ± 0.2	1.2 ± 0.3
AICAR	0 ± 0	0 ± 0	−0.2 ± 0.3	0.4 ± 0.3	0.7 ± 0.3	1.0 ± 0.5	0.9 ± 0.5	1.1 ± 0.6	0.9 ± 0.5	1.0 ± 0.4

### Effect of acute and chronic AICAR administration on key metabolites prior to and during hemorrhage

**Figure 4:**
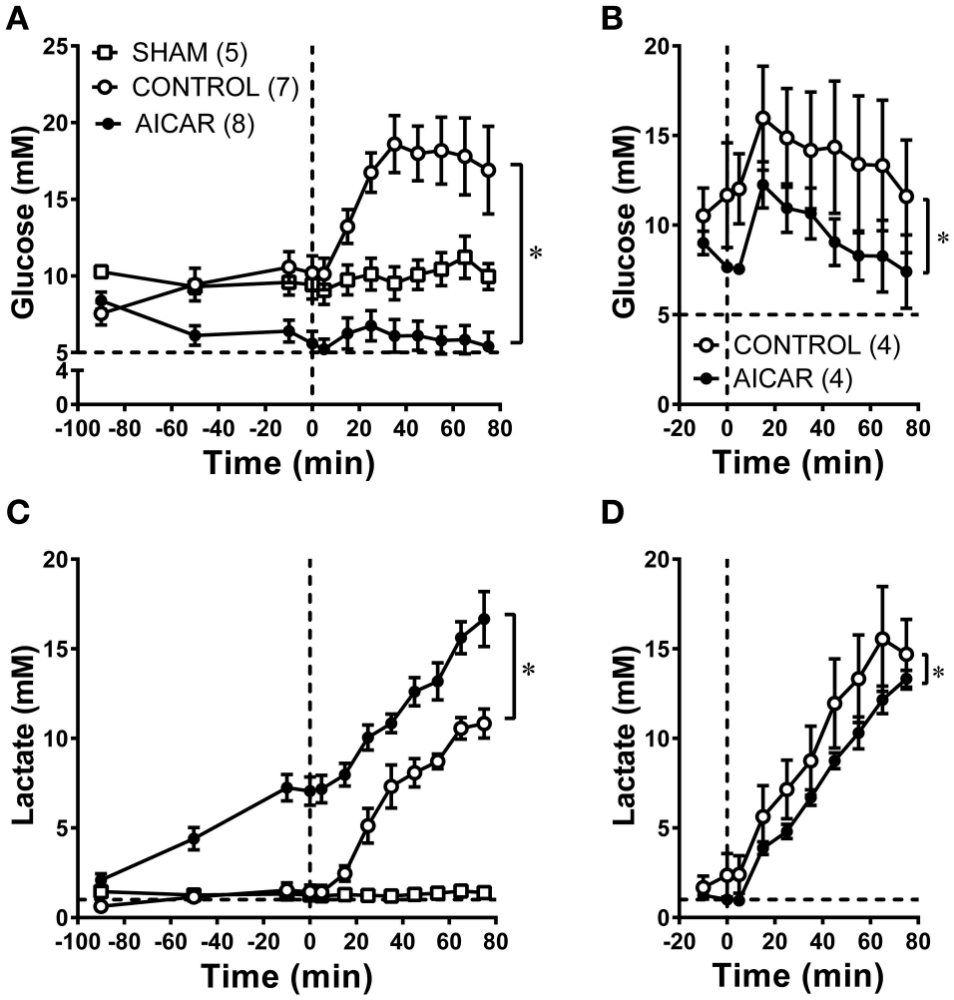
Comparison of arterial blood glucose **(A,B)** and arterial blood lactate **(C,D)**, levels at baseline and during hemorrhage in animals treated acutely **(A,C)** or chronically **(B,D)** with AICAR. ^*^P < 0.05; group effect—AICAR compared to control animals, both at baseline **(A,C)** and during hemorrhage **(A–D)**. Horizontal dashed lines represent physiological blood levels of glucose (5 mM, **A,B**) and lactate 1 mM, **(C,D)**; vertical dashed lines in **A–D** represent the onset of hemorrhage (see Section Methods).

All animals exhibited baseline arterial blood glucose levels above 5 mM (Figures 4A,B, dashed line), as might be expected from the combination of anesthesia and surgical stress. Blood glucose levels in animals acutely infused with AICAR (Figure 4A) were lower than those found in control (saline infused) animals just prior to hemorrhage (5.6 ± 0.8 vs. 10.2 ± 1.1 mM; Figure 4A, ANOVA significant group effect). As expected, control animals experienced a brisk rise in arterial glucose levels during hemorrhage (10.2 ± 1.1 to 18.6 ± 1.9 mM at 35 min following the onset of hemorrhage); these levels remained high throughout the hemorrhage period. This hyperglycemia was almost completely abolished in AICAR treated animals (Figure 4A, ANOVA group effect), which experienced only a modest increase (~1 mM) in blood glucose (5.6 ± 0.8 to 6.7 ± 1.0 mM at 25 min following onset of hemorrhage), followed by a reduction to pre-hemorrhage values. Arterial blood glucose was only slightly lower in AICAR chronically treated animals compared to vehicle controls prior to hemorrhage (Figure 4B). While hemorrhage induced a rise in arterial blood glucose in animals chronically treated with AICAR or vehicle, arterial blood glucose remained ~4–5 mM lower in rabbits pretreated with AICAR compared with vehicle treated animals (significant group effect, Figure 4B). Acute AICAR infusion was associated with significant increases in blood lactate concentrations prior to hemorrhage compared to vehicle infused control animals (Figure 4C; ANOVA group effect). Arterial blood lactate concentrations increased from 2.1 ± 1.0 to 7.1 ± 2.2 mM prior to the onset of hemorrhage. During the same period, control and sham animals demonstrated non-significant changes in blood lactate levels (controls − 0.6 ± 0.1 to 1.4 ± 0.4, sham 1.4 ± 0.3 to 1.3 ± 0.2 mM). Control animals had the expected increment in lactate levels to a peak of 10.8 ± 0.8 mM by the end of hemorrhage (Figure 4C). AICAR-infused animals had elevated arterial blood lactate which continued to increase to 16.7 ± 1.5 mM at the end of the hemorrhage period. Two-way ANOVA indicated a significant group effect (AICAR lactate levels significantly higher than controls; Figure 4C). Chronic AICAR infusion was associated with effects on blood lactate that were dramatically different than those observed in the acute AICAR infusion group (Figure 4D). Pre-hemorrhage lactate values were not significantly different between animals chronically infused with AICAR (1.0 ± 0.1 mM) vs. vehicle (2.4 ± 1.2 mM). Moreover, following the onset of hemorrhage, arterial blood lactate averaged 2–3 mM less in AICAR treated animals, with peak hemorrhage values of 15.6 ± 2.9 mM in controls, and 13.3 ± 0.5 mM in AICAR treated animals (Figure 4D, ANOVA group effect).

## Discussion

Key findings of this study are that AICAR administration (1) attenuated hemorrhage induced hyperglycemia and (2) lowered accumulated oxygen debt in the rabbit during moderate to severe hemorrhage. In addition, acute AICAR administration had an overall quantitatively larger effect on blood glucose levels, while chronic AICAR administration had a greater effect on curbing oxygen debt accumulation. Although acute AICAR administration was associated with a higher arterial blood lactate in the rabbit, the degree of lactate accumulation during hemorrhage was reduced in chronically AICAR treated compared to control animals.

Multiple studies support the role of AMPK as a central regulator of glucose metabolism (Gowans and Hardie, 2014). AICAR is a known activator of AMPK, and has shown promise in some forms of ischemia-reperfusion injury (Steinberg and Kemp, 2009). There have been several previous investigations of AICAR in the treatment of hemorrhage. AICAR enhances the whole animal oxygen consumption response during resuscitation seen with lactated Ringer's alone (Spiers et al., 1993), and reduces the infusion requirements after combined hemorrhage and lipopolysaccharide infusion (Fabian et al., 1996). AICAR also attenuates the intestinal permeability changes seen after hemorrhage; these effects were duplicated with adenosine infusion (Ragsdale and Proctor, 2000). In a rodent lethal hemorrhage model (Sonneborn and Rutten, 2011), AICAR extends survival time when given either before blood withdrawal or at the onset of low volume resuscitation. The survival benefits published by Sonnenborn and Rutten inspired us to examine the effects of AICAR on oxygen delivery and debt accumulation as a potential mechanism in improving survival. More recently, Basilia Zingarelli's laboratory has examined AICAR's role as an AMPK activator in hemorrhage and shown that AICAR can attenuate both myocardial and lung pathophysiology in murine hemorrhagic shock (Klingbeil et al., 2017; Matsiukevich et al., 2017).

Factors contributing to increases in blood glucose during hemorrhage include increased sympathetic outflow leading to hepatic (Kawai and Arinze, 1983) and muscle (Richter et al., 1982) glycogenolysis due to adrenoceptor activation, which also leads to an increased glucagon (Gerich et al., 1974) and decreased insulin secretion (Cerchio et al., 1973). While this immediate response may be adaptive, both early (Sperry et al., 2007) and persistent (Sperry et al., 2009) hyperglycemia seems to be associated with higher mortality and morbidity in critical illness. However, reduction of blood glucose with insulin is associated with the risk of severe hypoglycemia. Therefore, agents that lower blood glucose via insulin-independent pathways could be beneficial in trauma patients.

In the present study, the effect of AICAR on blood glucose reduction prior to hemorrhage was modest, and significant only in animals given AICAR acutely. A stronger effect was seen on the prevention of hyperglycemia following the onset of hemorrhage. Both of these effects may be mediated in part by activation of the alpha-2 isoform of AMPK, followed by increased glucose transport and GLUT4 protein expression (Steinberg and Kemp, 2009) via phosphorylation of TBC1D1 (Szekeres et al., 2012).

The effect of AICAR treatment on arterial blood lactate also depended on the mode of administration. When given acutely before hemorrhage, lactate levels rose to significantly higher levels than those seen in control animals before blood removal. This clearly represents aerobic lactate production, since hemodynamics including cardiac output were stable, and whole-body DO_2_ remained above the previously determined critical DO_2_ for this species (Lubarsky et al., 1995). Similar blood lactate changes have been noted in rats after a single ip injection of AICAR (Aschenbach et al., 2002). However, the dramatic increase in plasma lactate in the context of smaller decreases in plasma glucose following acute AICAR treatment was unexpected. It is possible that acute activation of AMPK disrupts the normal Randle (i.e., glucose-fatty acid) cycle by enhancing both glucose and fatty acid oxidation (Hue and Taegtmeyer, 2009). In such a state, excess pyruvate could not be efficiently converted by pyruvate dehydrogenase (already inhibited in some trauma states; Hue and Taegtmeyer, 2009) to acetyl-CoA. The excess would therefore be reduced to lactate. Finally, AICAR altered lactate clearance could contribute to the elevated plasma lactate levels secondary to AMPK mediated suppression of gluconeogenesis (Koo et al., 2005). Additional experiments will be necessary to address these mechanisms. It is not clear if lower acute dosing could have eliminated hyperglycemia during hemorrhage without acutely elevating plasma lactate levels. Chronic administration of AICAR was not associated with higher baseline lactate levels. While hemorrhage-induced hyperglycemia was attenuated or eliminated, the arterial blood lactate rose in rabbits treated acutely or chronically with AICAR. However, in acutely treated animals, the already elevated lactate continued to rise in parallel with vehicle treated controls (elevations of roughly 9 mM in both groups). Rabbits treated chronically with AICAR responded differently; arterial lactate levels were 2–3 mM less than in vehicle treated animals during hemorrhage, peaking at 13.3 ± 0.5 compared to 15.6 ± 2.9 mM. These differences in acute vs. chronic AICAR dosing may be due to effects of more prolonged AICAR administration on mitochondrial biogenesis (Komen and Thorburn, 2014), which was not assessed in these studies. These results are in contrast to those observed by Sonneborn and Rutten, who reported lower blood lactate following hemorrhage in AICAR treated animals (Sonneborn and Rutten, 2011). However, the dosing regimen described in that publication was different than in this study.

Oxygen delivery fell dramatically in both AICAR and vehicle treated animals following the onset of hemorrhage, whether treated acutely or chronically, due primarily to hemorrhage induced decreases in cardiac output. This led to nearly identical falls in oxygen consumption for animals treated acutely with either AICAR or vehicle animals early in hemorrhage. However, a slightly smaller fall in oxygen consumption in AICAR treated animals later in hemorrhage resulted in significantly smaller oxygen deficits. Animals chronically treated with AICAR had slightly smaller falls in oxygen consumption throughout hemorrhage and hence smaller deficits compared to animals chronically treated with vehicle (Tables 1, 2). This led to a larger (~54%) reduction in mean cumulative debt by the end of hemorrhage, despite changes in MAP and oxygen delivery that mirrored those seen in the acute animals. The smaller drops in oxygen consumption may have been in part due to greater peripheral oxygen extractions. This in turn could be due to a redistribution of cardiac output or enhanced mitochondrial function. For instance, AICAR has been shown to ameliorate mucosal (but not serosal) ileal ischemia following hemorrhage in swine (Ragsdale and Proctor, 2000).

The smaller oxygen debt in the face of larger arterial lactate values in the acutely treated AICAR animals in turn represents a dissociation between lactate and oxygen debt that may occur under some circumstances (Gladden, 2004), perhaps as a result of enhanced glucose transport. A lower oxygen debt could represent a potential mechanism for the longer survival times observed in AICAR treated rats following hemorrhage (Sonneborn and Rutten, 2011). Furthermore, the lower oxygen debt is a highly relevant finding, since the cumulative oxygen debt is the only variable that can consistently and quantitatively predict hemorrhage related morbidity and mortality in both human trauma patients and animal models of hemorrhage (Barbee et al., 2010).

AICAR is a cell-permeable nucleoside which can be phosphorylated by adenosine kinase to 5-aminoimidazole-4-carboxamide ribotide (AICA ribotide or ZMP), which activates AMPK (Wong et al., 2009). AICAR may act via other mechanisms as well (Guigas et al., 2009), and we did not intend to identify all of the mechanisms contributing to AICAR's actions in these experiments. However, gene knockdown (Shan, 2010) or knockout (Viollet et al., 2009) could be used to dissect the distinct contribution of AMPK activation following AICAR administration.

There are several limitations worth noting in this study. We utilized a controlled, pressure-clamped hemorrhage model in anesthetized animals, and AICAR was given as a pre-treatment. Giving AICAR as a pre-treatment obviates certain pharmacokinetic concerns (e.g., distribution and uptake of AICAR, metabolism to ZMP, activation downstream of AMPK α-1 vs. α-2 subunits, etc.), but is not as clinically relevant as resuscitation treatment. However, the results may still have relevance to treatment of ongoing hemorrhage or potential pretreatment in high risk surgical patients (Shoemaker and Belzberg, 1997) and show the potential of AMPK activation in modulation of oxygen debt and hence morbidity and mortality in shock. Future resuscitation studies are planned. We used right atrium sampling of central venous blood as a substitute for true mixed venous blood sampled from the pulmonary artery. This sampling location was used after initial attempts at pulmonary artery catheterization using a 5F thermodilution catheter led to hemodynamic instability. Central venous blood does not contain any venous return from the coronary sinus, and may not be fully mixed at all times, leading to sampling variability. While it is unlikely that right atrial sampling led to the significant differences in AICAR induced oxygen deficit and debt, it likely increased variability in the preparation. We are now refining the model to obtain true mixed venous sampling using a 4F pediatric monitoring catheter and are also in the process of developing an indirect calorimetry technique for measurement of oxygen consumption with less variability. However, the current reverse Fick technique for oxygen consumption determination could be adapted to unanesthetized animals following chronic aortic flow probe implantation to avoid the many confounding effects of anesthesia on hemorrhage responses (Lomas-Niera et al., 2005). We used only male rabbits for this study to simplify the experimental design. The association of sex and trauma is complex and has been the subject of many studies (Angele et al., 2008; Sperry and Minei, 2008). AMPK may mediate some of the effects of estrogen on the vasculature (Gayard et al., 2011), and both androgens and estrogens may regulate LKB1, an upstream kinase (Mcinnes et al., 2012). Therefore, the observed effect of AICAR on oxygen debt may not be identical in female rabbits, and additional experiments could address the interactions of sex steroids, AMPK, and hemorrhage.

In summary, we found that AICAR abolished the hyperglycemia and lowered the cumulative oxygen debt associated with hemorrhage in the anesthetized rabbit, possibly via activation of AMPK and downstream targets. These results suggest that activators of AMPK could play a role in decreasing morbidity and mortality associated with trauma via improved glucose control and lowering oxygen debt. The results strongly warrant additional studies to investigate effects of AICAR given as a resuscitation treatment.

## Author contributions

YH, AM, VL, GC, and YC—acquisition/analysis of data, revision of manuscript, final approval, agreement to be accountable for manuscript accuracy/integrity. PR—conception and design of project, analysis/interpretation of data, revision of manuscript, final approval, agreement to be accountable for manuscript accuracy/integrity. RB—conception and design of project, acquisition/analysis of data, drafting and revision of manuscript, final approval, agreement to be accountable for manuscript accuracy/integrity.

### Conflict of interest statement

The authors declare that the research was conducted in the absence of any commercial or financial relationships that could be construed as a potential conflict of interest.
